# A study of the oxidative processes in human plasma by time-resolved fluorescence spectroscopy

**DOI:** 10.1038/s41598-022-13109-0

**Published:** 2022-05-30

**Authors:** Tomasz Wybranowski, Blanka Ziomkowska, Michał Cyrankiewicz, Maciej Bosek, Jerzy Pyskir, Marta Napiórkowska, Stefan Kruszewski

**Affiliations:** Biophysics Department, Collegium Medicum of Nicolaus Copernicus University, Jagiellońska St. 13, 85-067 Bydgoszcz, Poland

**Keywords:** Biological fluorescence, Biophysics, Biomarkers

## Abstract

The aim of this study was to examine the usefulness of time-resolved fluorescence spectroscopy in the evaluation of the oxidative processes in human plasma. To investigate the impact of oxidative stress on the fluorescence of plasma, five studied markers (thiobarbituric acid-reactive substances, ischemia modified albumin, carbonyl groups, hydrogen peroxide, advanced oxidation protein products) were chosen as oxidative damage approved markers. Our method presents several advantages over traditional methods as it is a direct, non-time-consuming, repeatable, and non-invasive technique that requires only simple pre-treatment of samples without additional reagents and the sample size needed for analysis is small. In principle, each modification of the protein in plasma can be expected to modify its fluorescence properties and hence its lifetime or intensity. The study involved 59 blood donors with no evidence of disease. The research was conducted at excitation wavelengths of 280 nm and 360 nm, and emission was measured at wavelengths of 350 nm and 440 nm, respectively. Our results, although preliminary, suggest that the application of fluorescence measurements can be considered as an effective marker of oxidative stress. Regression analyses showed that a notable growth in fluorescence intensity at 440 nm and a simultaneous decrease in fluorescence intensity and mean fluorescence lifetime at 350 nm are associated with higher levels of oxidative stress.

## Introduction

The study, the results of which are presented in this paper, focused on a group of healthy people. According to the authors, the presented method opens up interesting new approaches and is the appropriate way to find a new marker of oxidative stress. Oxidative stress is defined as a disruption of the equilibrium between the generation of free radicals and the activity of antioxidant systems. The consequences of oxidative stress are modifications of the structure and function of proteins, nucleic acid damage, and lipid peroxidation. Oxidative stress is the background of many lifestyle diseases, so it’s important to find a sensitive indicator of undesired processes associated with it. In the pathological state, many activities of the organism are disturbed which results in the appearance of some substances hindering assessment of the level of oxidative stress. Furthermore, many oxidative markers do not correlate well with each other or do not properly reflect a state of oxidative stress^1^. In our opinion, if a new marker is significantly correlated with other markers in healthy groups it can be concluded that reflects the oxidative processes and is also appropriate for measuring oxidative stress in diseased states.

Proteins are likely to be major targets for free radicals, as a result of their abundance in plasma. There have been numerous in vitro studies showing damage to proteins under oxidation. As these modifications are likely to cause conformational and structural changes of the protein, the consequence of oxidation reactions may appear in the changes in fluorescence parameters of proteins. The emission of plasma protein is generally dominated by the tryptophan fluorescence and to a lesser extent by tyrosine. The contribution of phenylalanine to the fluorescence of plasma protein is negligible by low absorptivity and low quantum yield.

Time-resolved fluorescence spectroscopy is an important and widely used technique in physical sciences and is very useful for the study of protein–ligand interactions^2^. The time-resolved measurement reveals fluorescence intensity decay in terms of lifetimes. The high environmental sensitivity of the fluorescence lifetime can make it a complementary method to traditional fluorescence intensity measurements. Measurements at 280 nm excitation wavelength make it possible to examine the proteins, which have maximum absorption at 270–290 nm. It’s clear and confirmed that fluorescence lifetime is different for each patient and can contain information about their state. The lifetime and intensity of the fluorescence of plasma at 350 nm can contain information about the degree of oxidative damage of proteins since the signal of plasma fluorescence is connected mainly with the fluorescence features of amino acids (tryptophan, tyrosine, phenylalanine). Free radicals lead to the oxidation of these amino acids, forming dityrosine containing cross-linked protein products, generating carbonyl moieties, nitration of tyrosine residues and other environmental modifications^3^. It should be noted that other amino acids, and their changes can also influence the fluorescence of plasma due to the efficient transfer of electronic energy between amino acids. For now, these processes have not been described well. Some of the energy transfer can be enhanced or weakened during oxidative stress. Measurements at 360 nm excitation wavelength allow the decrease in the contribution of different components, especially proteins and free amino acids. The emission at 440 nm is probably related to advanced glycosylation end products (AGEs) or advanced lipid peroxidation end products (ALEs)^4,5^. The characteristic fluorescence of AGEs is similar to that of Maillard food products and depends on the reaction between reducing sugars and amino groups (non-enzymatic glycosylation of proteins)^6^. An excess of AGEs in the blood is characteristic not only of diabetes but is also associated with most pathological states in organisms^7^. Fluorescence of ALEs is created primarily by reactions of aldehydes with amino groups of protein (Schiff bases)^8–10^. Many studies show that the formation of AGEs and ALEs is related to oxidative stress^11^. Although some of the mechanisms involved in the increase in fluorescence emission have been studied, especially in vitro processes, the fluorescent compounds have not been yet fully characterised. Many other biomolecules may also contribute to the overall fluorescence measured at 440 nm.

To investigate the impact of oxidative stress on the fluorescence properties of human serum albumin (HSA), some initial experiments were performed. As a result of the artificial oxidation of HSA (by hydrogen peroxide, UVC irradiation, chloramine T and Hypochlorous acid), glycation (by glucose and fructose), temperature denaturation (up to 70 °C) and reaction with aldehydes (Malondialdehyde, MDA), the following was noticed: a decrease in both the mean fluorescence lifetime and the intensity of fluorescence a wavelength of 350 nm, and an increase in the mean fluorescence lifetime and the intensity at a wavelength of 440 nm (data not shown). These results convinced us to use the fluorescence properties of plasma to measure oxidative stress in people. It was expected that different levels of oxidative stress cause different changes in the fluorescence properties of plasma. Proof of this dependence could allow the use of the fluorescence parameters as a new marker of oxidative stress. This study is based on an empirical correlation between fluorescence parameters (intensity and lifetime) and five biomarkers namely: thiobarbituric acid-reactive substances (TBARS), ischemia modified albumin (IMA), carbonyl groups (CO), hydrogen peroxide (H_2_O_2_), and advanced oxidation protein products (AOPP). To the best of our knowledge, this is the first study analysing the fluorescence lifetime of plasma as a marker of oxidative stress. As this alteration of protein function appears to be of medical relevance, this study may have an important value. Diseases influence the modification of proteins due to oxidative stress and this redox state may influence the fluorescence of plasma. The proposed optical method provides a possibility of a quick, non-invasive study of biological blood plasma samples with high repeatability of measurements. Some chemical methods have been developed for the measurement of specific kinds of protein modifications, however, the diagnostic relevance of the markers used is limited because they do not give a holistic view. The fluorescence of plasma can reflect all protein changes and can be considered as a global marker.

## Materials and methods

The human blood from healthy people was received from the Local Blood-Donation Center (Bydgoszcz, Poland). We included 59 patients, 19 women, and 40 men between the ages of 20 and 43 in this study. An 8 ml blood sample was taken for examination from each patient. Blood samples were added to standard sterile polystyrene tubes containing EDTA and then centrifuged at 4000 rpm for 5 min. Plasma was collected and divided to separate tubes for each measurement. Then, all measurements were performed within 2 h. The procedures were described below.

Carbonyl groups were determined by reaction with 2,4-dinitrophenylhydrazine (DNPH) leading to the formation of stable dinitrophenyl (DNP) hydrazone adducts, which were detected spectrophotometrically at 375 nm. The Sigma Aldrich (MAK094) assay kit was used according to the manufacturer’s recommendations for plasma.

The Fluorimetric Hydrogen Peroxide Assay Kit from Sigma Aldrich (MAK165) was used to find the level of H_2_O_2_. Sample preparation and measurement were made in accordance with the manufacturer’s protocol of determination. This kit utilises a peroxidase substrate that generates a fluorescent product at 590 nm after reaction with hydrogen peroxide.

The level of AOPP was determined by measuring absorbance at 350 nm according to the modified method described for the first time by Witko-Sarsat^12^. Briefly, the reactant mixture for the AOPP assay contained 1.875 ml of 0.2 M citric acid and 25 µl of 1.16 M potassium iodide. 1.9 ml of this mixture was then added to 100 µl of the test sample and the absorbance was recorded after 30 min. In comparison with the original method, citric acid was used instead of acetic acid. This modified method is characterised by greater stability over time^13^.

The concentration of aldehydes (TBARS) was determined by measuring absorbance at 532 nm. Briefly, the reactant mixture for TBARS contains 20% trichloroacetic acid (TCA) and 0.375% thiobarbituric acid (TBA) diluted in 40 mM thiobarbituric acid (HCL) and 0.01% butylated hydroxytoluene (BHT) diluted in ethanol. 2 ml of this mixture was then added to 1 ml of the plasma sample and boiled for 60 min at 95 °C. The precipitate was pelleted by centrifugation at 4000×*g* at room temperature for 10 min and then the absorbance of the supernatant at 532 nm was recorded.

Measurement of IMA was measured using the colorimetric method developed by Bar-Or et al.^14^. Briefly, 200 µl of plasma was mixed with 50 µl of 0.1% cobalt chloride and incubated for 10 min to ensure sufficient cobalt albumin binding. Then, dithiothreitol (DTT) solution (50 µl, 1.5 mg/ml) was added to enable reaction with unbound cobalt. After incubation for 2 min, 1 ml of 0.9% NaCl was added to stop binding between cobalt and albumin and then the absorbance at 470 nm was recorded.

The time-resolved spectrofluorometer Life Spec II (Edinburgh Instruments Ltd, United Kingdom) with the sub-nanosecond pulsed EPLED^®^ diode emitting light at wavelengths of 280 or 360 nm was used in order to measure the fluorescence intensity (INT) and lifetime (FLT) of the diluted plasma. Plasma samples were 25-fold diluted in phosphate-buffered saline (PBS). The exposure time of samples was 30 s at excitation of 280 nm and 2 min at 360 nm. Fluorescence measurements of the plasma were made at wavelengths of 350 and 440 nm, respectively. The obtained data were treated with deconvolution analysis taking into account the instrumental response function. mFLT value was calculated as the weighted average of fluorescence lifetimes obtained from the three-exponential model of fluorescence decay. As averaging weights, the contributions of individual components (areas under decay curves) to the total fluorescence were taken. The appropriate number of exponents was determined on the basis of the analysis of Chi-square (χ^2^) statistics and visual assessment of residual plots. The subject of interest was the mFLT of the sample, therefore the model was simplified as much as possible in order to maintain computational stability.

The preliminary step of the statistical analysis was the Shapiro–Wilk test of normality of the distribution of measured parameters. Due to the non-normality of the part analysed variables the dependencies were determined by Spearman’s rank correlation coefficients (r values). The differences were compared with the Mann–Whitney U test and were considered significant at p < 0.05.

### Ethical approval

The study was conducted according to the guidelines of the Declaration of Helsinki, and approved by the Ethics Committee Nicolaus Copernicus University in Toruń, Collegium Medicum in Bydgoszcz (KB 116/2011). Written informed consent was obtained from the patients.

## Results and discussion

There have been many studies that reported a connection between oxidative stress and the development of several diseases. It is usually noted that the degree of plasma oxidation increases with aging and not much differs by gender^15–19^. In the presented study, no significant correlations between age and markers of oxidative stress were observed (Table 1; correlation coefficient r ranging from 0.02 to 0.25; p > 0.05). The reason for this may be that our study group consisted mainly of young and middle-aged healthy people. Most studies analysing the link between age and oxidative stress have focused on the entire population, including people with medical conditions and the elderly. There were also no significant differences by gender in our test group (p > 0.05). Some findings suggest that greater longevity observed in women than men is associated with more efficient antioxidant activity^20^. It is well known that there is a difference between some oxidative markers in comparison to control subjects but there are usually no data showing a correlation between the studied markers, especially in healthy people. In this work, for the collected plasma samples the biomarker levels of oxidative stress were established and the correlations between them were calculated and featured in Table 1.

**Table 1:** Correlation coefficients between approved markers and age.

Markers	AOPP	H_2_O_2_	IMA	CO	TBARS	Age
AOPP	1.00	0.53***	0.66***	0.28*	0.24	0.25
H_2_O_2_	0.53***	1.00	0.43**	0.29*	0.27*	0.11
IMA	0.66***	0.43**	1.00	0.18	0.41**	0.15
CO	0.28*	0.29*	0.18	1.00	0.47***	0.02
TBARS	0.24	0.27*	0.41**	0.47***	1.00	0.07

Oxidation parameters: advanced oxidation protein products (AOPP), hydrogen peroxide (H_2_O_2_), ischemia modified albumin (IMA), carbonyl groups (CO), thiobarbituric acid-reactive substances (TBARS). Correlation coefficients and significance between markers were calculated according to Spearman’s method: *p < 0.05, **p < 0.01, ***p < 0.001.

As expected, significant positive correlations (p < 0.05) were revealed between all these biomarkers, which point to their involvement in similar mechanisms of oxidative stress. AOPP was found to be highly correlated with IMA (r = 0.66, p < 0.001) and H_2_O_2_ (r = 0.53, p < 0.001). Also, between IMA and H_2_O_2_ a relatively high correlation was observed (r = 0.43, p < 0.01). There was also a high positive correlation between TBARS and carbonyl groups (r = 0.47, p < 0.001), but the TBARS level does not appear to be very highly correlated with AOPP and H_2_O_2_. One can assume that a new marker of oxidative stress should be strongly correlated with the markers mentioned above.

For the collected plasma samples fluorescence decay curves were measured at 350 nm and 440 nm for excitation at 280 nm and 360 nm, respectively. Measurement of the fluorescence lifetime using the time-correlated single-photon counting (TCSPC) method is based on the assumption that the statistical distribution of time intervals between excitation and emission obtained for individual fluorophores is asymptotically (after a large number of excitation-emission cycles) consistent with the distribution of fluorescence lifetime of a large number of fluorophores excited at once. Each TCSPC cycle begins with a sub-nanosecond excitation laser pulse and ends with the detection of a single photon. The time between these events is accurately measured. The histogram of the obtained intervals reflects the fluorescence decay of the entire sample and is analysed by fitting the multi-exponential function to extract the amplitudes and fluorescence lifetimes^21–23^. More specifically, the result of the convolution of the multi-exponential model and the impulse response function is here compared with the recorded decay curve. It should be noted that the individual lifetimes obtained from the fitting analysis do not reflect the individual sample components.

Intrinsic plasma fluorescence at 350 nm is due to the aromatic amino acids, mainly tryptophan. Fluorescence of phenylalanine has a very low quantum yield, and emission of tyrosine in native proteins is often quenched. According to the literature, the emission of L-tryptophan in water originates from two forms of the tryptophan formed in the excited state, with fluorescence lifetimes equal to 0.4 and 2.8 ns^24^. Our results indicated that the fluorescence lifetimes of L-tryptophan in PBS were equal to 0.6 and 3.1 ns with a share of 9% and 91% respectively (χ^2^ = 1.1). The monoexponential fitting does not give good results in the chi-square sense (χ^2^ = 1.62). However, the intrinsic fluorescence decay due to tryptophan in HSA is known to be three-exponential. The third fluorescence lifetime is the result of the interaction between the tryptophan residue and the surrounding microenvironment in protein^25–27^.

**Figure 1:**
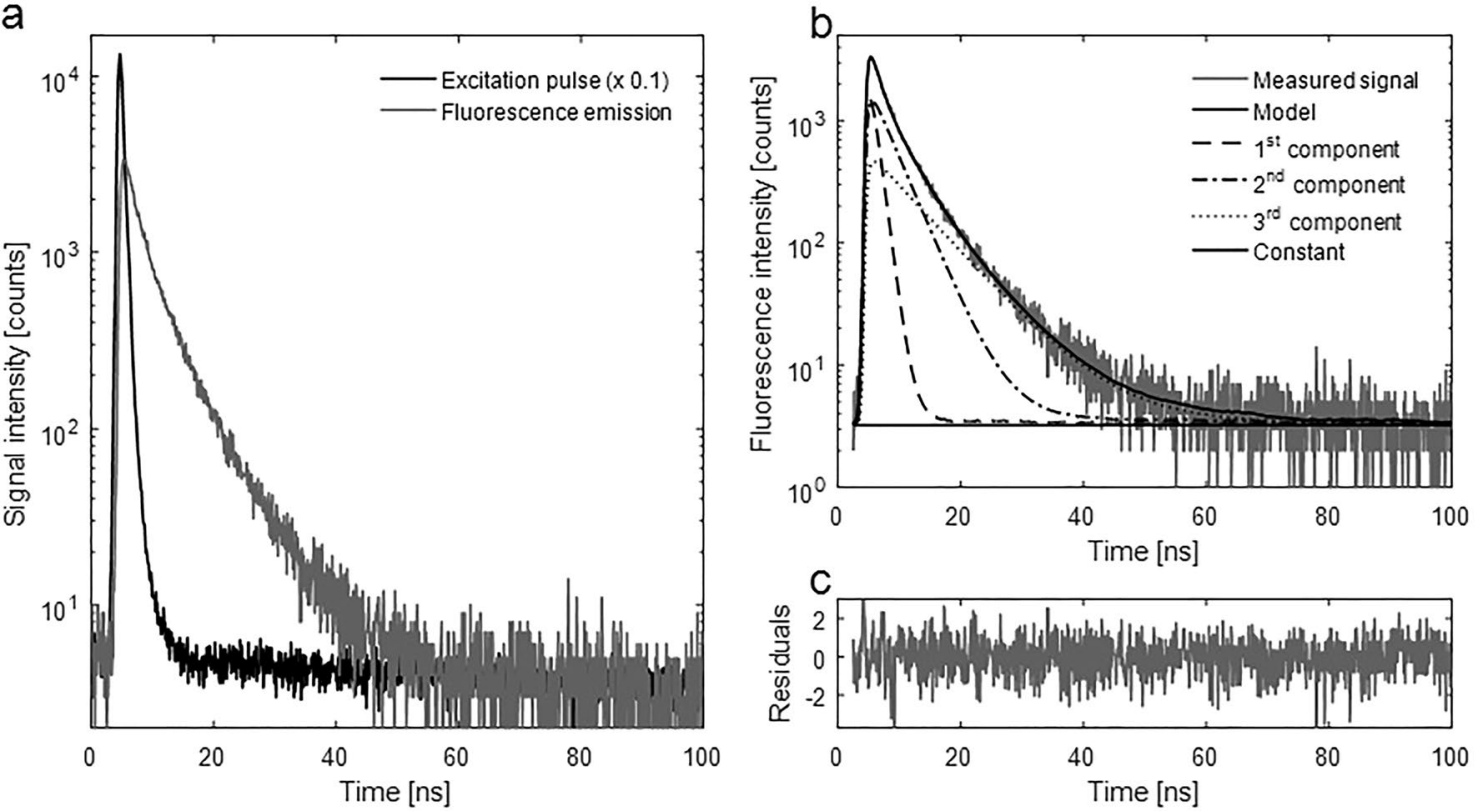
The decomposition of fluorescence lifetime (FLT) decay on particular components: (**a**) the signal intensity of plasma at 350 nm, (**b**) the model of three exponential fluorescence decay (χ^2^ = 1), (**c**) the residuals of the model.

Figure 1 presented the decomposition of fluorescence lifetime decay on particular components for a plasma sample at 350 nm emission. The fluorescence decay curves of the plasma samples deviated from a single-exponential function; therefore, in order to obtain the correct fluorescence decay characterization, it was necessary to use the multiexponential fluorescence decay model. The three-exponential model provided the best fit on the basis of the smallest Chi-square (χ^2^). The three obtained fluorescence lifetimes depend on the intrinsic characteristics of the tryptophan and also on the local environment, aggregation, interactions with other molecules, and also oxidation of amino acids in plasma proteins. For this reason, in this work, the authors analysed only a mean FLT (mFLT) weighted by the fractional contribution of each component to the steady-state intensity calculated from three-exponential models. The application of the mFLT test simplifies the numerical analysis. In the case of emission at 440 nm, the fluorescence of plasma originates from many different components such as collagen, reduced nicotinamide dinucleotide (NADH), flavin adenine dinucleotide, AGEs and ALEs. The real distribution of FLTs in such a system is probably much more complex, especially when one realizes that different conformations of the same specimen may exhibit different deactivations of the excited state that would require a much more extensive (and difficult to implement) model. The results of our previous study revealed that the increase in the concentration of hydrolysed collagen added to plasma reduced the fluorescence lifetime at 450 nm depending on the degree of hydrolysis^28^. Aldehydes react with various amino acids in protein and then form several fluorescent compounds in a process called protein lipoxidation^29,30^. Our experiment showed that adding MDA to the HSA and plasma solution increased the mFLT depending on the incubation time. In addition, different aldehydes form different fluorescent products with proteins in the plasma, which is confirmed by slightly different fluorescence lifetimes. Various fluorescent products are also formed after protein glycation^31^. It is well known that the fluorescence lifetime of NADH changes when it binds to its cofactors^32^. In summary, there are too many fluorophores at an emission of 440 nm to relate the fluorescence lifetimes obtained in the deconvolution of fluorescence decays, to specific compounds. Therefore, it was also decided to consider only the mean fluorescence lifetime (mFLT) calculated from the three-exponential model.

The correlations between the fluorescence parameters (intensity and mFLT) and approved markers (described above) and age are shown in Table 2.

**Table 2:** Correlations between fluorescence parameters of plasma and approved markers and age.

Markers	INT 440	mFLT 440	INT 350	mFLT 350
AOPP	0.42**	0.07	− 0.34**	− 0.17
H_2_O_2_	0.23	0.13	− 0.38**	− 0.17
IMA	0.22	0.04	− 0.40**	− 0.38**
CO	0.03	0.06	− 0.12	− 0.34**
TBARS	− 0.03	0.10	− 0.36**	− 0.54***
age	0.20	0.15	− 0.15	0.11

Fluorescence parameters: intensity at 440 nm (INT 440), mean fluorescence lifetime at 440 nm (mFLT 440), intensity at 350 nm (INT 350 nm), mean fluorescence lifetime at 350 nm (mFLT 350). Univariate correlation coefficients and significance between parameters were calculated according to Spearman’s method: *p < 0.05, **p < 0.01, ***p < 0.001.

**Figure 2:**
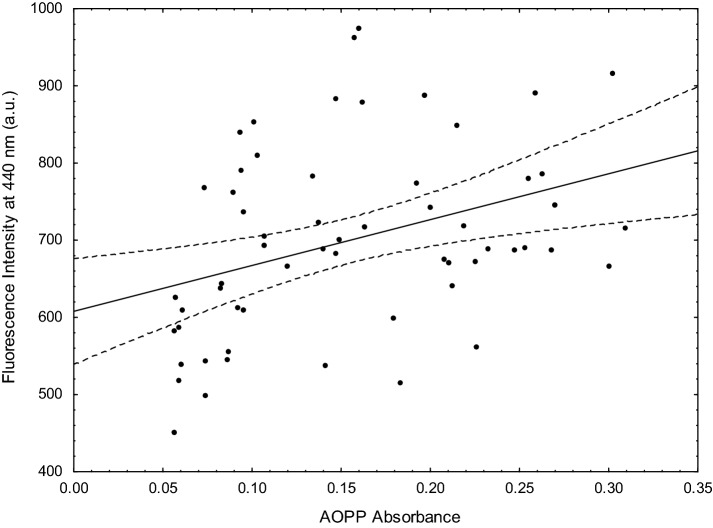
Dependence between fluorescence intensity at 440 nm and AOPP absorbance in healthy plasma donors. The dashed lines represent the 95% confidence intervals for the regression line.

As one can see, the changes in fluorescence intensity at 440 nm were the most strongly correlated with AOPP (Fig. 2), mFLT at 350 nm could be applied to predict the level of aldehydes (TBARS) and (with less probability) IMA and CO, while the intensity of fluorescence measured at 350 nm could be related to the level of all analysed markers except CO.

The increase of fluorescence intensity of plasma at 440 nm upon oxidative stress has been raised in some papers. The fluorescent compounds at 440 nm are formed as a result of the reactions between sugars and/or lipid peroxidation products (aldehydes) and amino groups from proteins, respectively, AGEs or ALEs^8,9^. As AGEs and ALEs are mostly derivatives of oxidatively modified albumin and their levels increase when the antioxidant defence is highly disturbed, fluorescence intensity at 440 nm can be revealed particularly in people with well documented involvement of oxidative stress in their etiopathogenesis. Some researchers revealed a significant correlation between AGE-peptide levels measured by enzyme-linked immunosorbent assay (ELISA) and fluorescence intensity at 440–460 nm, especially in diabetic patients^33^. Intensified processes of glycation (AGEs) and oxidation (AOPP) are closely linked, and many studies show high correlations between these two markers^34,35^. Furthermore, Chelh claims that dityrosine-aldehydes complexes are also highly fluorescent^35^. AOPP is closely correlated with levels of dityrosine^12^. So, as was expected, in parallel to the increase of fluorescence at 440 nm an increase in AOPP in our study was observed (r = 0.42, p < 0.01). Similarly, Kalousová showed a high correlation between fluorescence at 440 nm and AOPP in diabetes mellitus and hemodialysed patients^36^.

There have been some studies considering the fluorescence intensity at 400–460 nm in plasma in several disease states. Munch et al. showed a significant increase in fluorescence intensity in hemodialysis patients in comparison with the control subjects (111.9 × 10^3^ vs 30.7 × 10^3^ arbitrary units, p < 0.0001)^5^. Sebeková et al. obtained similar results in children with renal disease^37^. Recently, Sergio Raposeiras-Roubín examined the prognostic value of fluorescent AGEs in the context of acute coronary syndrome^38^. In their study, high fluorescent AGEs levels were associated with more follow-up events. Another study’s results showed that the levels of fluorescent intensity were significantly increased in the plasma from patients with Type 2 diabetic patients presenting with vascular complications^39^.

There was no correlation between fluorescence intensity at 440 nm and TBARS (r =  − 0.03). There is no doubt that aldehyde is an active modifying agent of proteins both in vitro and in vivo^8^. Burcham showed that in vitro incubation of bovine serum albumin (BSA) with the toxic lipid peroxidation product (malondialdehyde-MDA) resulted in a time- and concentration-dependent increase in fluorescence intensity at 450 nm, while MDA itself does not fluoresce^40^. These results were in good agreement with those obtained in human serum albumin (HSA) by us (data not shown). However, our studies of the plasma of healthy patients showed that this modification occurs without the formation of covalent protein adducts that emit fluorescence at 440 nm. The mechanisms of these reactions are poorly understood. Taking into account numerous, often very complex, interactions in the body, an organism could develop a repairing process to prevent the creation of more advanced aldehyde-proteins adducts. It is very likely that the creation of ALEs is efficiently prevented by antioxidants. On the other hand, the fluorescence of ALEs could be masked by the fluorescence of AGEs. It is difficult to estimate which product is mainly attributed to the enhancement of fluorescence in plasma during oxidative stress. Moreover, the level of TBARS does not have to be correlated with the amount of ALEs in the plasma due to the interaction of aldehydes with proteins which prevents their reaction with TBA. It should be also noted that TBA reacts not only with MDA but also with many other compounds such as other aldehydes, carbohydrates, amino acids and nucleic acids interfering in the TBA assay and resulting in considerable overestimation. Different results were obtained in rats with streptozotocin-induced diabetes mellitus^41^. This study revealed a very high correlation between fluorescence at 450 nm and lipid peroxides both in non-diabetic and diabetic rats. However, the analysis of lipid peroxides in that study was based on the ability of lipid peroxides to convert iodide to iodine, which in the assay mixture consequently reacts with iodide to form I_3_.

The decrease of fluorescence parameters (both intensity and mFLT) of plasma at 350 nm is not clear and has not been examined in detail. However, changes in the fluorescence of some compounds (e.g., albumin or tryptophan itself) have been used in some works to monitor physicochemical changes in plasma proteins. It is well known that in vitro oxidation of proteins by reactive oxygen species (ROS) and other free radicals generates some modifications of proteins influencing a decrease in fluorescence intensity at 350 nm. For example, Sutherland and Reza showed that the incubation of healthy serum or BSA with hypochlorous acid (HOCl) or sodium hypochlorite solution resulted in a decrease in protein tryptophan fluorescence^42,43^. In vitro studies showed that HOCl leads to the generation of AOPP, similar to those existing in blood^34^. So, it is not surprising that the fluorescence intensity at 350 nm was correlated with AOPP in our healthy subjects (r =  − 0.34, p < 0.01). Hydrogen peroxide (H_2_O_2_) as a prooxidant also induces oxidative stress. Our results showed that an increasing level of H_2_O_2_ was weakly associated with an increase of the fluorescence intensity of plasma at 440 nm (r = 0.23, not significant) and slightly with a decrease at 350 nm (r =  − 0.38, p < 0.01).

**Figure 3:**
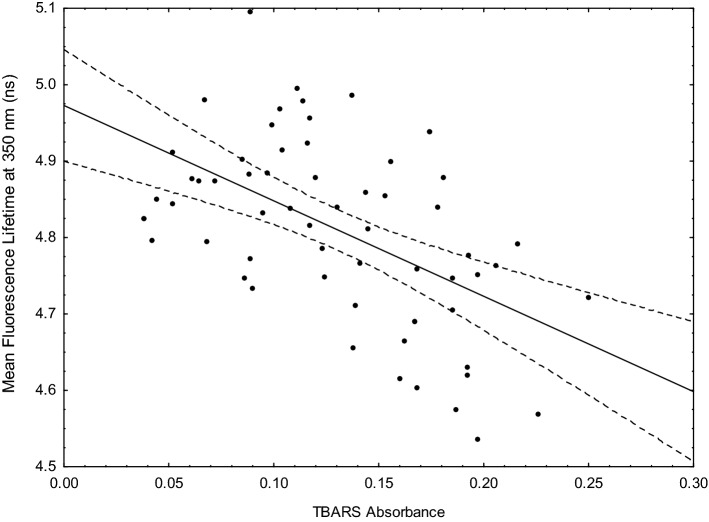
Dependence between mean fluorescence lifetime (mFLT) at 350 nm and TBARS absorbance at 532 nm in healthy plasma donors. The dashed lines represent the 95% confidence intervals for the regression line.

As is apparent from Table 2 and Fig. 3 a linear relationship between the decrease in mFLT at 350 nm and the level of lipid oxidations, as measured by the TBARS absorbance, was observed (r =  − 0.54, p < 0.001).

These results showed that aldehydes, created by the oxidation of lipoprotein, react with the amino acids of proteins, influencing fluorescence at 350 nm. In the in vitro study, we confirmed a decrease in fluorescence parameters at 350 nm after adding MDA to HSA (data not shown). The studies conducted by Burcham and Kuhan also showed a reduction in fluorescence intensity at 350 nm^40^. Giessauff et al. suggest that the fluorescence of tryptophan is connected with the lipid peroxidation process in the low-density lipoprotein (LDL)^44^.

The IMA marker has been found to be sensitive and an early biochemical marker of ischemic heart disease but is now used as an important marker of oxidative stress. The increase in IMA levels reflects the decrease in albumin-cobalt binding. According to the results of our previous study, the increase in levels of oxidative stress causes a decrease in the binding associate constant of some drugs^45^. IMA can reveal both early and late modifications of albumin due to quite a good correlation with all studied approved markers. The highest correlation between IMA and fluorescence parameters was observed at 350 nm. The correlation coefficients are r =  − 0.4 and − 0.38 for fluorescence intensity and mean fluorescence lifetime, respectively.

Non-significant correlations between markers and fluorescence lifetime at 440 nm were established in our study. On the basis of this data, it can be concluded that during oxidative stress the amounts of fluorescent AGEs or ALEs change but the structures of these products remained unaltered.

A different implementation of fluorescent properties of plasma was developed by Wu et al.^46–48^. In their work, the potential causes or implications of oxidative stress (smoking, hypertension and reduced renal function or occupational exposure to irritant cleaning products) were correlated with the fluorescence intensity of plasma at 440 nm after mixing with ethanol/ether. Furthermore, data obtained from Rebholz’s study indicates that an elevated level of this marker is associated with chronic kidney disease status and its severity^49^. On the other hand, this plasma fluorescent oxidation product was not correlated with the risk of estrogen receptor-negative breast cancer or erectile dysfunction^50,51^. Some researchers have tried to use other organic solvents (more apolar) instead of a mixture of ethanol and ether. Their works show that the fluorescence of these products after extraction in chloroform can be a new oxidative marker in Alzheimer’s disease, but further detailed study is needed^52^. In another study, the negative association between this marker and the level of selenium was established^53^.

## Conclusion

The conducted research and analyses showed that the applied time-resolved fluorescence spectroscopy method can be useful in oxidative stress research. One can say that the increase in fluorescence intensity at 440 nm and the simultaneous decrease in fluorescence intensity and mean fluorescence lifetime at 350 nm are associated with an increase in oxidative stress. Time-resolved spectroscopy was successfully correlated with the traditionally approved methods commonly used to determine oxidative stress. On the other hand, the fluorescence methods have some advantages over the traditional approaches. The fluorescence measurements performed in this study were not very technically advanced. However, further work with plasma from donors in a diseased state is required to establish this spectroscopic technique as a tool for estimating oxidative stress, as it is believed that proteins alter during the development of pathologies. Intensive research should be still conducted on the possibility of using the fluorescence of plasma as useful indicators for the diagnosis, predicting, and monitoring of diseases. It should be noted that by measuring fluorescence intensity and mean fluorescence lifetime the specific proteins responsible for specific disease mechanisms can also be examined.

## Data Availability

The datasets generated during and analysed during the current study are available from the corresponding author on reasonable request.
